# Carbon Nanomaterials From Metal-Organic Frameworks: A New Material Horizon for CO_2_ Reduction

**DOI:** 10.3389/fchem.2020.573797

**Published:** 2020-10-14

**Authors:** Xiaoxu Xuan, Songying Chen, Shan Zhao, Joon Yong Yoon, Grzegorz Boczkaj, Xun Sun

**Affiliations:** ^1^Key Laboratory of High Efficiency and Clean Mechanical Manufacture, Ministry of Education, School of Mechanical Engineering, Shandong University, Jinan, China; ^2^National Demonstration Center for Experimental Mechanical Engineering Education, Shandong University, Jinan, China; ^3^Shandong Key Laboratory of Water Pollution Control and Resource Reuse, School of Environmental Science and Engineering, Shandong University, Qingdao, China; ^4^Department of Mechanical Engineering, Hanyang University, Ansan, South Korea; ^5^Department of Process Engineering and Chemical Technology, Faculty of Chemistry, Gdańsk University of Technology, Gdańsk, Poland

**Keywords:** carbon dioxide CO_2_ reduction, nanomaterials, MOFs, green chemistry, carbon catalysts

## Abstract

The rise of CO_2_ in the atmosphere, which results in severe climate change and temperature increase, is known as the major reason for the greenhouse effect. Reducing CO_2_ to value-added products is an attractive solution to this severe problem, along with addressing the energy crisis, to which the catalysts being employed are of vital importance. Due to their high porosity and tunable compositions, metal-organic frameworks (MOFs) show great potential in energy conversion systems. By thermal or chemical treatment methods, the MOFs are easily turned into MOF-derived carbon nanomaterials. The much higher level of conductivity enables MOF-derived carbon nanomaterials to be employed in CO_2_ conversion processes. The present review, discusses the state of the art of MOF-derived carbon nanomaterials in CO_2_ electrochemical, photocatalytic, and thermal reduction applications. The corresponding reaction mechanisms and influence of various factors on catalyst performance are elaborated. Finally, the deficiencies and recommendations are provided for future progress.

## Introduction

The catalytic reduction of carbon dioxide (CO_2_) to value-added products is an effective way of alleviating the severe environmental problem of global warming (Ran et al., 2018; Li et al., 2019). Great efforts have been devoted to developing advanced CO_2_ conversion systems, including CO_2_ electrochemical (Tripkovic et al., 2013; Ma et al., 2017; Wang et al., 2017), photocatalytic (Crake et al., 2017; Pipelzadeh et al., 2017; Cardoso et al., 2018; Li et al., 2019), and thermal catalytic reduction systems (Chaemchuen et al., 2019; Lin et al., 2019; Zeng et al., 2020). For these CO_2_ conversion systems, the key factor which impacts the efficiency and conversion rate is the CO_2_ reduction catalyst (Samanta et al., 2012; Zheng et al., 2017; Yaashikaa et al., 2019).

Metal-organic frameworks (MOFs), as an emerging category of porous materials, have attracted great interest due to their unique physicochemical properties, such as their highly specific surface area, tunable porosity, and controllable functionality (Liu et al., 2010; Aiyappa et al., 2019; Bhadra et al., 2019). Therefore, MOFs have been widely applied in various energy conversion and storage systems, such as gas separation and storage (Furukawa and Yaghi, 2009; Salehi and Anbia, 2017), drug delivery (Cai et al., 2019a,b; Safaei et al., 2019), biosensors (Zhang et al., 2016; Qiu et al., 2019), heterogeneous catalysis, and CO_2_/N_2_ conversion (An et al., 2017; Luo et al., 2019). However, because of their unsatisfactory electrical conductivity and stability, it greatly hinders their application in CO_2_ reduction processes. By thermal or chemical treatment, the pristine MOF composites can be converted into MOF-derived carbon materials with embedded metal nanoparticles or metal oxides (Qian et al., 2017; Bhadra et al., 2019; Shi et al., 2019). These MOF-derived carbon materials generally combine the advantageous physicochemical properties of the pristine MOF (porosity and tunable chemical compositions) and carbon/metal (high conductivity, active metal sites) which offers more possibilities for catalysis. The strategies of design of carbon materials derived from MOFs can not only improve the charge transportation abilities of materials and shorten the CO_2_ molecules diffusion path, but also create more active sites on the materials.

In this mini review, we highlight recent advances in the application of MOF-derived carbon materials for CO_2_ reduction processes, including electrochemical, photocatalytic, and thermal reductions. Recent progress and development of MOF-derived carbon materials for CO_2_ conversion were also discussed. Additionally, at the end of this review, we give a brief perspective of MOF-derived carbon materials in CO_2_ conversion.

## Fabrication of MOF-Derived Carbon Materials

**Figure 1:**
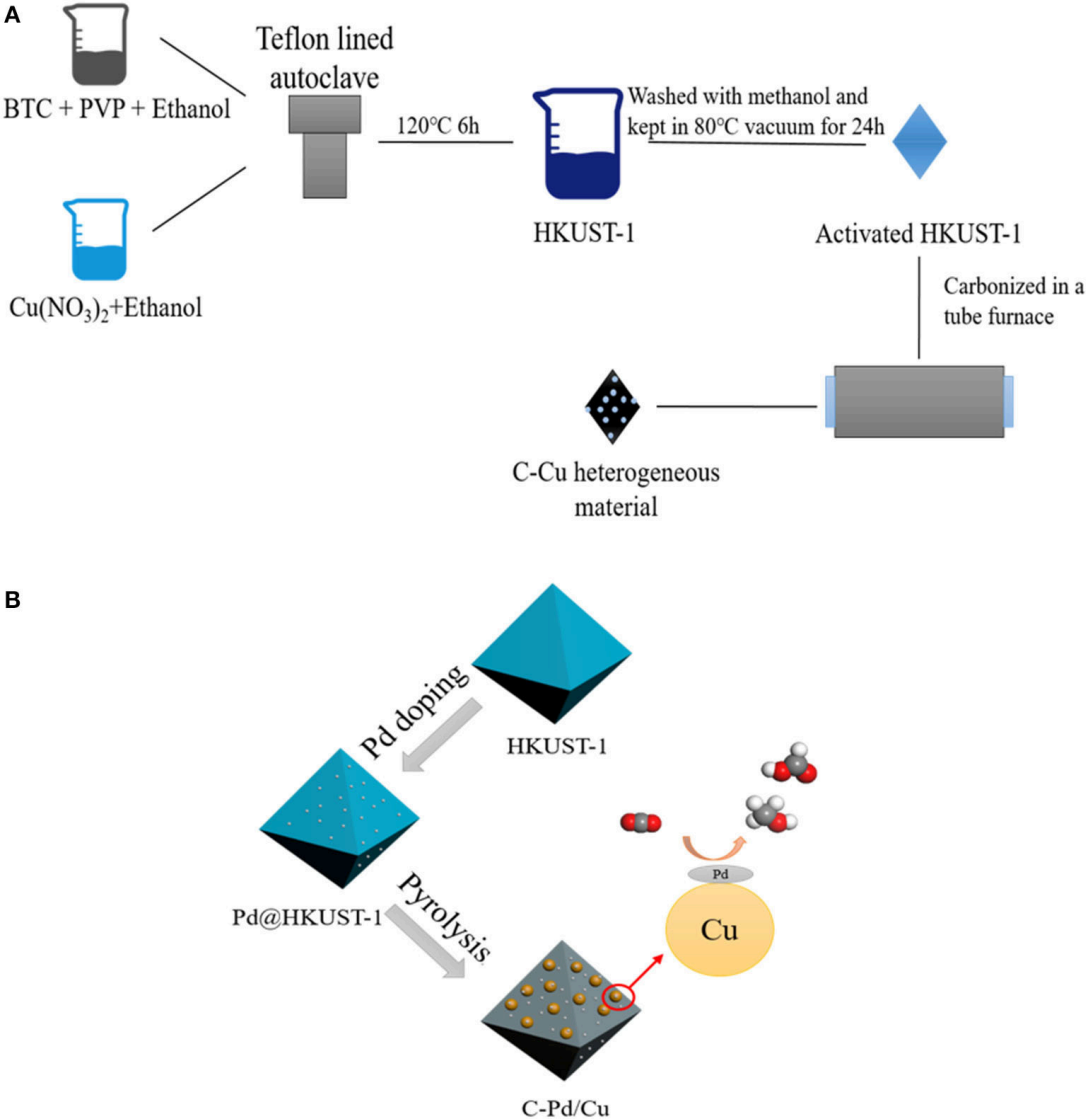
Typical synthetic process of MOF-derived carbon materials (HKUST-1 as the pristine MOF): self-templating method **(A)** and external-templating method **(B)**. (BTC, 1,3,5-Benzenetricarboxylicacid; PVP, polyvinyl pyrrolidone; HKUST-1, a kind of Cu MOF).

MOFs can be assembled by metal ions/clusters and organic linkers through coordination bonds by hydrothermal (Chen et al., 2020), microwave (The Ky et al., 2020), electrochemical (Vehrenberg et al., 2020), ultrasonic (Zhao et al., 2020), or hydrodynamic cavitation methods (Aiyappa et al., 2019; Kim et al., 2020; Sun et al., 2020a,b). Generally, the MOFs possess special properties, including porous structures and tunable chemical compositions which enable the desirable design of MOF-derived composites with high catalytic activity. In the first report of MOF-derived carbon materials (Liu et al., 2008), carbon materials were synthesized by thermal transformation of pristine MOFs. Due to the special structure of MOFs (a topology in which metal atoms are connected by ligands), metal oxides, metal phosphides, metal chalcogenides, and metal carbides can be synthesized *in-situ* in the carbon matrix. Researchers have studied the impact of synthesis conditions on the physicochemical properties of MOF-derived carbon materials. The results show that under the high-temperature conditions of pyrolysis treatment, the structure of pristine MOFs tended to collapse, and their porosities were damaged, leading to the aggregation of metal atoms in the carbon matrix. In addition, recent studies (Khalid et al., 2018; Bhadra et al., 2019) indicated that by choosing appropriate pristine MOFs and controlling the synthetic conditions subtly, the morphologies of pristine MOFs can be reserved after the pyrolysis process. The high-temperature treatment of pristine MOFs offers an effective way of precisely controlling the shape, size, and structure and at the same time maintains the materials' functionalities in one step. Generally, the most used pyrolysis synthetic methods of MOF-derived carbon materials can be categorized into two types: self-templating and external templating methods. In the self-templating method, only pristine MOF is pyrolyzed, while in the external templating method, pristine MOF, as well as some external templates (including metal nanoparticles, graphene, silica, and metal oxides) are pyrolyzed. A typical scheme of the self-templating method and external-templating method is shown in Figure 1. Besides the above-mentioned synthetic methods, the *in-situ* growth of MOF crystals on carbon materials is also an effective and easily performed method to synthesis MOF-derived carbon materials.

## MOF-Derived Carbon Materials in CO_2_ Electrochemical Reduction

**Figure 2:**
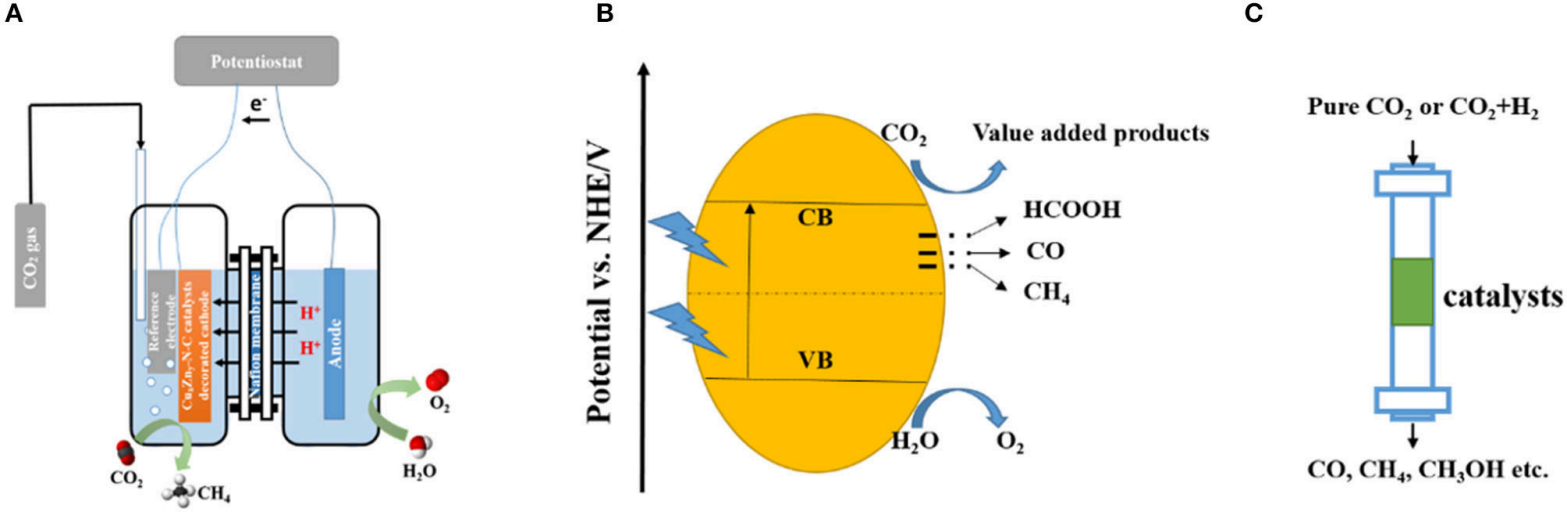
Scheme of CO_2_ reduction systems: electrochemical reduction system **(A)**, photocatalytic reduction system **(B)**, and thermal catalytic reduction system **(C)**.

As a promising approach to produce value-added products, the electrochemical reduction of CO_2_ has attracted many researchers since it was first reported by Hori et al. (1985). Double-cell reactors, with a Nafion membrane used to separate the cells, are widely used for this process (Figure 2A).

Typically, the oxidation reaction occurs on the anode of the system, water is oxidized to produce oxygen and protons, the proton then goes through the Nafion membrane to take part in the CO_2_ reduction reaction which happens on the cathode. Generally, the electrochemical reduction of CO_2_ involves different reaction pathways, which lead to various products (Equations 1–6). By tuning the compositions and functionalities of the electrochemical reduction catalysts, the selectivity and CO_2_ conversion rate can be largely improved.

(1) $$\begin{array}{l} CO_{2}+8H^{+}+8e^{-}\to CH_{4}+2H_{2}O \end{array}$$

(2) $$\begin{array}{l} 2CO_{2}+14H^{+}+14e^{-}\to C_{2}H_{6}+4H_{2}O \end{array}$$

(3) $$\begin{array}{l} CO_{2}+6H^{+}+6e^{-}\to CH_{3}OH+H_{2}O \end{array}$$

(4) $$\begin{array}{l} 2CO_{2}+12H^{+}+12e^{-}\to C_{2}H_{5}OH+3H_{2}O \end{array}$$

(5) $$\begin{array}{l} 3CO_{2}+18H^{+}+18e^{-}\to CH_{3}CH_{2}CH_{2}OH+5H_{2}O \end{array}$$

(6) $$\begin{array}{l} 3CO_{2}+18H^{+}+18e^{-}\to CH_{3}CH\left(OH\right)CH_{3}+5H_{2}O \end{array}$$

As MOF-derived carbon materials possess the properties of high conductivity, large porosities, and highly active reaction centers, they are excellent catalysts for high-efficiency CO_2_ reduction.

Copper is reported to have high activity and selectivity in CO_2_ electrochemical reduction. Fabricating copper or copper oxides containing carbon materials based on MOFs offers a facile method to synthesize highly active CO_2_ electrochemical reduction catalysts (Xuan et al., 2019, 2020). The most used pristine Cu MOF in the fabrication of CO_2_ electrochemical catalysts is HKUST-1. Oxide-derived Cu/carbon (OD Cu/C) catalysts were synthesized by the carbonization of HKUST-1. Zhao et al. fabricated OD Cu/C catalysts with temperatures of 900, 1,000, and 1,100°C. The obtained materials exhibited high selectivity in CO_2_ reduction to alcohol products, and the highest selective CO_2_ reduction to ethanol was achieved on OD Cu/C-1000 at an overpotential of 190 mV. The electrochemical catalytic activity of this catalyst was attributed to the synergistic effect between the highly dispersed copper and the matrix of the porous carbon (Zhao et al., 2017). Cheng et al. used the external template method to synthesize a Pd nanoparticle-doped carbonized HKUST-1 catalyst (C-Pd/Cu). The noble metal-copper embedded in the carbon matrix structure enabled the fast transportation and easy adsorption of CO_2_ molecules, facilitating CO_2_ hydrogenation (Cheng et al., 2019b).

Due to its special surface charge density distribution which leads to high CO_2_ adsorption ability, metal-N sites containing MOF-derived carbon materials have recently attracted attention (Cheng et al., 2019a). Bao's group has devoted large efforts to this area. Fe-N and Ni-N active sites containing ZIF-derived carbon materials were successfully synthesized by direct carbonization of pristine Fe/Ni-doped ZIF. Ammonia treatment was also employed on the Fe-N-containing catalyst to improve the specific area and mesopore areas, thus effectively boosting CO_2_ reduction (Yan et al., 2018b). A coordinatively unsaturated Ni-N active site containing a ZnNi ZIF-derived porous carbon catalyst was also fabricated and employed in CO_2_ electrochemical reduction. The highest CO Faradaic efficiency of 98% was achieved. Density functional theory (DFT) calculations revealed that the CO_2_ reduction reaction was favored more than the hydrogen evolution reaction over Ni-N sites embedded in the porous carbon structure (Yan et al., 2018a).

## MOF-Derived Carbon Materials in CO_2_ Photocatalytic Reduction

Photocatalytic CO_2_ reduction is a process in which the catalyst absorbs sunlight radiation and creates electron-hole pairs to evoke CO_2_ molecules, generating value-added products (Figure 2B). In this energy converting process, three fundamental steps are needed: (1) sunlight absorption by the catalyst to create electro-hole pairs; (2) generation and migration of redox equivalents; and (3) reduction and oxidation reactions with the redox equivalents at the catalytic active centers (Zhang and Lin, 2014).

Due to their unique structures, tunable compositions, and porosities, MOF-derived carbon materials can contain photosensitizers and catalytic active centers in a single solid. A number of recent studies have demonstrated that MOF-derived carbon materials show high catalytic activity in CO_2_ photocatalytic reduction. Hu et al. reported an HKUST-1-derived hollow C-Cu_2−x_S nanotube/g-C_3_N_4_ composite for CO_2_ photoreduction with H_2_O vapor. During the CO_2_ photocatalytic reduction process, the carbon coat in the catalyst acted as an electron reservoir, which facilitated electron-hole pair separation. The optimized C-Cu_2−x_S@g-C_3_N_4_ acted as a photocatalyst. The reactivity and selectivity were boosted to 1062.6 μmol g^−1^ and 97% respectively, which were much higher than those when bare Cu_2_S or g-C_3_N_4_ was applied under the same conditions (Hu et al., 2019).

*In*-*situ* growth of MOF crystals on carbon materials is an effective method to synthesize MOF-derived carbon materials that possess both the properties of porous MOF and carbon materials. Liu et al. fabricated a g-C_3_N_4_/ZIF-8 composite by a simple *in-situ* heterogeneous deposition method. The designed hybrid photocatalyst not only inherited the broadened optical properties of g-C_3_N_4_, but also had a high CO_2_ adsorption capacity due to the porous structures of ZIF-8. Herein, the g-C_3_N_4_/ZIF-8 photocatalyst exhibited much higher activity for CO_2_ photocatalytic reduction, reaching 0.75 μmol h^−1^g^−1^ under 365 nm light radiation. This study provided a promising method for the easy synthesis of MOF-derived carbon materials (Liu et al., 2017).

MOF-derived carbon materials offer the possibility of tuning the size of active sites in the photocatalyst. Mu et al. successfully synthesized a series of C-BMZIF with a rhombic dodecahedral structure. The Zn and reduced Co presented in the carbon matrix could prevent Co active sites from aggregation. They came up with the idea that the size of Co active sites could be regulated by adjusting the Zn/Co ratio, thus influencing the compositions of the resulting products (Mu et al., 2018).

## MOF-Derived Carbon Materials in CO_2_ Thermal Catalytic Reduction

CO_2_ thermal catalytic reduction is an approach in which high temperature and pressure are applied to realize the conversion of CO_2_ into value-added products (Figure 2C). Compared with the above-mentioned methods to convert CO_2_ into value-added products, the CO_2_ thermal catalytic reduction method has a longer history. Nowadays, various approaches to CO_2_ thermal conversion have been widely used in industrial-scale applications. Generally, industrial-scale CO_2_ thermal catalytic reduction approaches can be divided into two types: pure CO_2_ decomposition and CO_2_ conversion with a co-reactant. As the CO_2_ thermal catalytic reduction process involves high temperature and high pressure, therefore, catalysts with high stability are required. The carbon matrix structure enables MOF-derived carbon material to endure the high temperature in CO_2_ thermal catalytic reduction and at the same time offers more anchoring dots for the CO_2_ molecules.

A robust and easily collected pyrolyzed bimetallic Zn/Co ZIF catalyst was successfully synthesized by Chaemchuen et al., and it showed high catalytic activity for the cycloaddition of CO_2_ into epoxides. The metal dispersion and catalytic properties of the catalyst were improved by the Co species hybrid with N species in the carbon matrix wall (Chaemchuen et al., 2019).

Lu et al. pyrolyzed ZIF-67 at a temperature of 700°C to fabricate a cobalt-based nonprecious metal catalyst for CO_2_ hydrogenation. The Mott-Schottky effect at the metal-support interface in the catalyst caused an electron transfer over the Schottky barrier. The first-principle mechanistic study revealed that the Co embedded in the carbon matrix could increase CO_2_ activation during the reaction (Lu et al., 2019). Lin et al. presented a facile synthesis of hierarchical Ni@C spheres with Ni nanoparticles confined in carbon shells for CO_2_ methanation under relatively low temperatures. The hollow and porous structures of the catalyst afforded a high surface area and isolated more active sites for CO_2_ methanation, therefore, resulting in high activity and superior selectivity in the CO_2_ thermal catalytic reduction (Lin et al., 2019).

## Conclusions and Perspectives

In this mini review, the application of MOF-derived carbon materials in CO_2_ reduction was summarized. MOF-derived carbon materials inherit the properties of high specific surface area, porous structures, and tunable compositions from pristine MOF. Furthermore, their charge transportation ability, electron-hole separation ability, and stability are improved comparing with pristine MOF. Though MOF-derived carbon materials have been widely used in the field of CO_2_ reduction, there still remain problems to be solved:

1. The reaction mechanisms of the CO_2_ electrochemical reduction in the aqueous phase electrolyte on MOF-derived carbon materials are still not clear. *In*-*situ* detecting methods should be utilized to further clarify the reaction pathways of CO_2_ reduction for various products.
2. The porous structure of the carbon matrix in MOF-derived carbon materials is unstable and prone to collapse during the pyrolysis and reduction reaction process. Substituting the internal carbon matrix with external carbon templates and building more robust MOF-derived carbon materials could be a solution for this issue.
3. The cost of MOF-derived carbon materials is high compared with traditional catalysts. To satisfy the need of MOF-derived carbon material applications in the industrial scale CO_2_ reduction process, large scale MOF-derived carbon material synthetic methods should be developed to lower the cost and improve the efficiency.

## Author Contributions

XS, JY, and SC contributed the conception of the study. XX produced and wrote the article. XS, GB, and SZ edited the article. All authors contributed to the article and approved the submitted version.

## Conflict of Interest

The authors declare that the research was conducted in the absence of any commercial or financial relationships that could be construed as a potential conflict of interest.
